# Supplementary material for: Differentially Expressed Genes in Cardiomyocytes of the First Camelized Mouse Model, Nrapc.255ins78 Mouse
Source: Genes (Basel). 2025 Jan 24;16(2):142. doi: 10.3390/genes16020142 (PMC11855364; doi:10.3390/genes16020142)
Supplement: Supplementary file 1 [file genes-16-00142-s001.zip › genes-3431183-supplementary.pptx]

## Slide 1
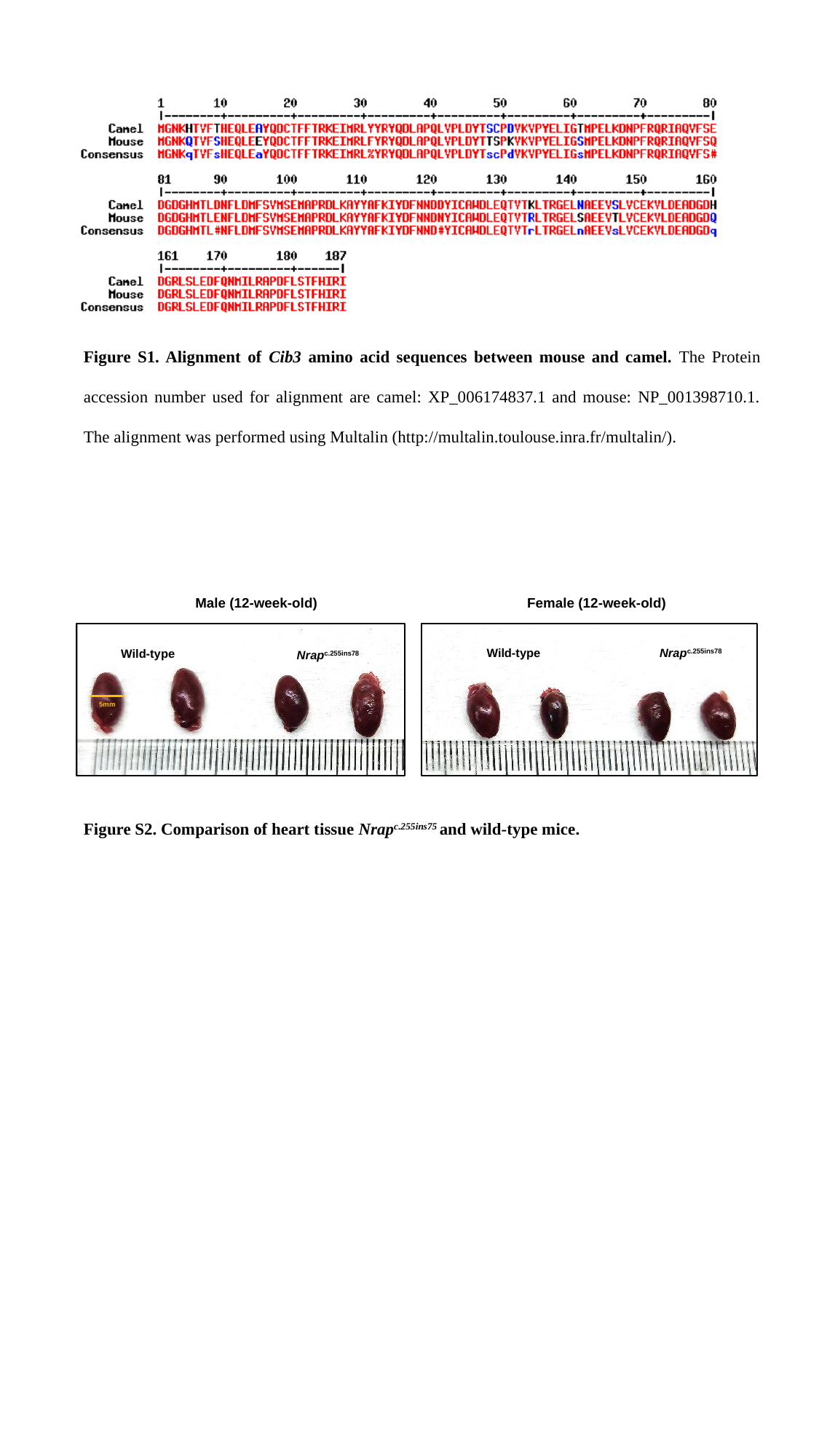

Figure S1. Alignment of Cib3 amino acid sequences between mouse and camel. The Protein accession number used for alignment are camel: XP_006174837.1 and mouse: NP_001398710.1. The alignment was performed using Multalin (http://multalin.toulouse.inra.fr/multalin/).
Male (12-week-old)
Female (12-week-old)
Wild-type
Nrapc.255ins78
Wild-type
Nrapc.255ins78
5mm
Figure S2. Comparison of heart tissue Nrapc.255ins75 and wild-type mice.
